# Urban Metabolism of Food-Sourced Nitrogen among Different Income Households: A Case Study Based on Large Sample Survey in Xiamen City, China

**DOI:** 10.3390/foods10112842

**Published:** 2021-11-17

**Authors:** Li Xing, Tao Lin, Xiongzhi Xue, Jiakun Liu, Meixia Lin, Yu Zhao

**Affiliations:** 1 College of the Environment and Ecology, Xiamen University, Xiamen 361102, China; lxing@iue.ac.cn (L.X.); xzxue@xmu.edu.cn (X.X.); 2 Key Laboratory of Urban Environment and Health, Institute of Urban Environment, Chinese Academy of Sciences, Xiamen 361021, China; mxlin@iue.ac.cn; 3 Department of Human Geography and Spatial Planning, Faculty of Geosciences, Utrecht University, 3584 CB Utrecht, The Netherlands; j.liu5@uu.nl; 4 State Key Laboratory of Urban and Regional Ecology, Research Center for Eco-Environment Sciences, Chinese Academy of Sciences, Beijing 100085, China; yuzhao1@rcees.ac.cn

**Keywords:** urban metabolism, structural equation model, household food consumption, food-sourced nitrogen, environmental emissions

## Abstract

Food consumption is fundamental for urban households if they are to sustain production and daily life. Nitrogen resulting from food consumption has significantly contributed to pollutant emissions in urban ecosystems. Taking Xiamen city, a rapid urbanizing area of southeast China as a case study, we evaluated the food-sourced nitrogen consumption of households based on a large simple onsite questionnaire survey, as well as differences between households in the consumption of plant-based and animal-based foods. A material flow analysis (MFA) was conducted to simulate the urban metabolism of food-sourced nitrogen and environmental emissions among different income groups. The impacts of household attributes, plant-based food consumption, and animal-based food consumption on environmental nitrogen emissions were examined with a structural equation model (SEM). Our results show that the surveyed households’ diets were more plant-based and less animal-based. Aquatic products and livestock were the source of 43.7% of food-sourced nitrogen, and 84.5% of the food-sourced nitrogen was discharge into the environment through direct discharge and waste treatment. Soil, water, and air emissions accounted for 62.8%, 30.1%, and 7.1% of the food-sourced nitrogen, respectively. Household income, household size, and household area are all associated with accelerating increases of nitrogen emissions released into the environment, though middle-income group households have the highest food-sourced environmental nitrogen emissions. On this basis, we discuss how to better manage the urban metabolism of food-sourced nitrogen, so as to improve urban household consumption, lower nitrogen emissions, and improve food security.

## 1. Introduction

Nitrogen is a major component of proteins, which are necessary for the existence of human beings and all other living organisms [1]. With worldwide urbanization, nitrogen flows and metabolic processes within cities are growing rapidly due to human activity [2,3,4,5,6,7,8,9]. Cities depend on nitrogen input, distribution, and output by human-dominated processes to maintain their functioning. This is commonly known as urban nitrogen metabolism [10,11]. Though essential, these processes have adverse impacts on the soil, water and atmosphere, and result in enhanced greenhouse effects [12,13], photochemical smog [14,15], water quality degradation [16,17,18], loss of soil nutrients [19,20], and biodiversity loss [21,22]. Urban nitrogen metabolism research has attracted increased attention in regional sustainable development [23].

Food is to nitrogen as energy is to carbon [24]. Food consumption is fundamental for urban residents to sustain their production and daily life. Simultaneously, population density [25], dietary changes [26], the food supply–demand ratio [27], and waste treatment facility development [28,29] at the urban scale can affect nitrogen flows and cycling in urban areas. It is the food consumption of households, however, that has the biggest impact on nitrogen flows in urban ecosystems. For instance, household nitrogen fluxes are influenced by dietary patterns, which account for 40% of total nitrogen inputs [30]. Long et al. determined the driving factors, including nutrient intake, household attributes, educational level, and health conditions as sources of the multiple footprints (including nitrogen) associated with household food consumption [31]. With respect to income, a higher household income may cause a greater environmental footprint (carbon dioxide, pollutants, water and energy) [32,33,34]; this relation can be seen to hold at the national level for nitrogen footprints [35]. Furthermore, the Chinese government has identified increased household consumption as a key driving force of economic development [36]. Hence, urban household food consumption among different incomes groups has become a prime target of environmental management and security.

Nitrogen footprints vary considerably between products. Animal-based food imposes the greatest environmental burdens [37], while plant-based food tends to require significantly fewer resources [38,39]. Animal-based products cause significantly higher nitrogen losses, even 10 times more than plant-based products [40]. There are potential benefits in replacing animal-based products with nitrogen efficient alternatives [41,42], which might conflict with nutritional considerations. Quantitative analysis of different food-sourced nitrogen consumption in urban households will help to understand the dynamics and trajectories of nitrogen fluxes in the urban ecosystem as a whole and to solve the environmental pollution problems resulting from nitrogen use. Material flow analysis (MFA) is a frequently used method to evaluate stocks and flows of substances [43], and it has been used to estimate nitrogen metabolism, as well as the combined impacts, from the perspective of cities [5,44]. These analyses have focused on the nitrogen input and output of whole cities, without specifically addressing the human-related differences among household groups within cities. The existing literature has not made use of relevant information about urban household attributes, such as income and building age, to see how environmental nitrogen emissions might be shaped by household dietary patterns.

In this study, we attempt to quantitatively stimulate the urban metabolism of food-sourced nitrogen among different income households and explore the impacts of household attributes on environmental nitrogen emissions. To achieve this goal, our study was designed as follows: (i) analyze the surveyed households’ food-sourced nitrogen consumption per capita, and animal/plant-based differences in this consumption, and the differences in household consumption across six income groups in 2011; (ii) apply the MFA method to estimate the dynamic flow paths of the nitrogen elements through the urban ecosystem, including food consumption, human digestion, waste treatment, and environmental emissions into air, water and soil; and (iii) use the SEM method to quantitatively analyze the impact factor of environmental nitrogen emissions produced by households. The conclusions drawn in this paper could help guide strategies for improving the urban metabolism of food-sourced nitrogen among households with different incomes, as well as food security and sustainable nitrogen cycling.

## 2. Materials and Methods

### 2.1. Study Area and Survey Sites

Xiamen, a coastal city in southeast Fujian Province, was one of the first “Special Economic Zones” in China [4]. From then on, Xiamen has experienced rapid urbanization and its urban population has grown at an amazing speed [45]. Its regional GDP increased from 16.47 billion yuan (comparable GDP) in 1980 to 599.95 billion yuan in 2019. Simultaneously, the urbanization ratio increased significantly from 35% to 89.2%. The population and households of Xiamen City reached 4.29 million persons and 0.82 million households. Per capita urban disposable income and consumption expenditure reached 59,018 yuan and 38,442 yuan respectively. However, the release of pollutants from urban metabolism into the environment, namely, environmental emissions, was exacerbated [3]. For example, the residential sewage annually discharged and the domestic garbage volume manufactured in Xiamen City went up from 152.72 million m^3^ and 0.47 million t in 2002 to 399 million m^3^ and 2.54 million t in 2019 [46].

**Figure 1:**
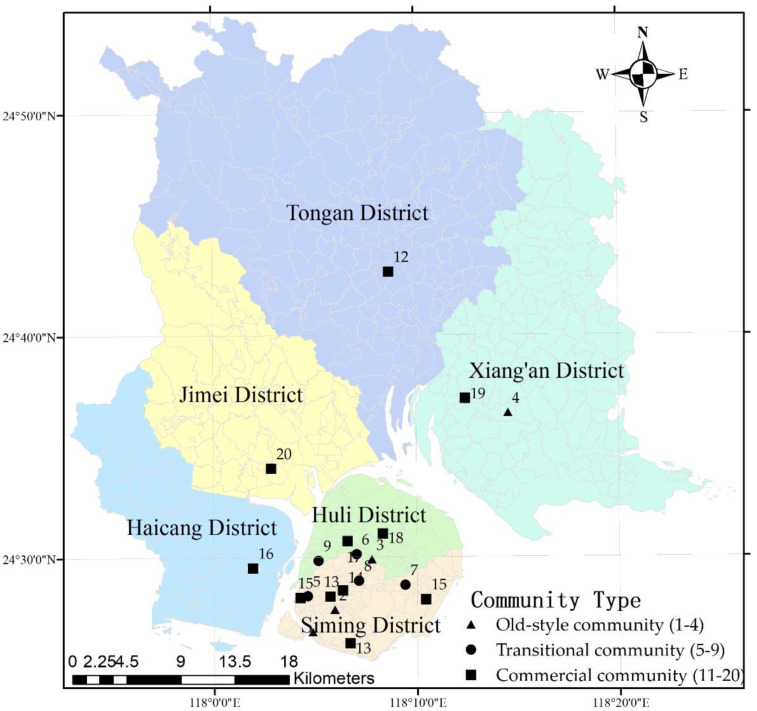
Distribution of surveyed communities in Xiamen City, China.

Xiamen has a land area of 1699.39 km^2^ and a sea area of 390 km^2^. The downtown area is located on Xiamen Island, including Huli District and Siming District, and the off-island districts are mainly peri-urban areas, comprising Xiang’an District, Tongan District, Haicang District, and Jimei District. According to the multi-objective spatial sampling model [47,48], community age and the stage division of housing system reform in China [49], 20 typical communities, including four old-style communities, five transitional communities and 11 commercial communities, were identified as the survey sites (see Figure 1). The onsite questionnaire surveys were conducted in the targeted communities in 2011; 503 questionnaires were completed by door-to-door interview. There were 500 questionnaires which met all the information needed for our study, distributed in above 20 communities on average.

### 2.2. Survey Design and Data Processing

In this study, full data for environmental nitrogen emissions due to household food consumption and influencing factor analysis were obtained from the questionnaires. Our questionnaire consisted of two latent variables: household attributes and household food consumption. The observed variables for each latent variable are shown in Table 1. Household food consumption in our study was classified according to the variables of plant-based food consumption and animal-based food consumption. Household size, household income, household structure, housing area, and building age were considered to be potential influencing factors among household attributes. Due to the low nitrogen content in edible oils, alcoholic beverages and tea, we took grain, vegetables, fruits, sugar, and pastry as potential influencing factors with respect to plant-based nitrogen consumption. Livestock, eggs, aquatic products, and milk were considered to be potential influencing factors with respect to animal-based nitrogen consumption. It ought to be noted that we only quantified urban household consumption and did not include data for out-of-home consumption (e.g., at restaurants and canteens).

Only partial questionnaire variables were quantitative (e.g., household income) while other variables were qualitative (e.g., household structure). However, all household attribute observed variables were converted into ordinal variables so as to facilitate statistical analysis in SPSS 22.0 and AMOS 22.0. The transformation standards and occurrence frequencies are shown in Table 2. SEM is a statistical method which combines statistical data with qualitative causal hypotheses to test and estimate causal relations. It integrates factor analysis and path analysis [50]. Factor analysis allows for seeing whether observed variables and latent variables (which cannot be directly observed) have a causal relationship and was used to determine the main influencing factors. Path relationships between endogenous latent variables, such as household attributes, and exogenous latent variables, such as urban household environmental nitrogen emissions, were applied to indicate the latent variables operating within an integrated framework.

### 2.3. Methods

#### 2.3.1. Nitrogen Content in Household Food Consumption

We calculated household food-sourced nitrogen production based on food consumption per capita, the nitrogen content coefficient of food, and household size. It was calculated as follows:

(1) $$HW_{i}=P_{i}\times Wp$$

(2) $$Wp=\overset{n}{\underset{j}{\sum }}w_{pj}$$

(3) $$w_{pj}=w_{j}\times p_{j}$$

where *HW_i_* represents the total nitrogen of food consumption in household *i*; $P_{i}$ is the population in household *i*, that is, household size; Wp is the nitrogen content of per capita food consumption in household *i*; $w_{pj}$ refers to the nitrogen content of food *j* consumption per capita; *n* is the category of household food consumption, *n* = 9; $w_{j}$ is the amount of food *j* consumption per capita. $p_{j}$ is the coefficient of the nitrogen content in food *j*, which hinges on the protein content according to the “2014–2018 China Mainland Food Balance Sheets” by the FAO. Its value is equal to the protein content multiplied by 0.16 [51] (see Table 3).

#### 2.3.2. Nitrogen Flow Path and Environmental Emissions Calculation

Material flow analysis (MFA) was used to quantify the food-sourced nitrogen flows of surveyed households in Xiamen City, China [52]. In this study, we concentrated on dynamic flow paths of the food-sourced nitrogen in the urban ecosystem, covering food consumption, human digestion, waste treatment, and environmental emissions. Food consumption was the only nitrogen input in the urban ecosystem. The sink had two parts: one part into the residents’ bodies through digestion–absorption, and the other into soil, water, and the air as environmental emissions through direct discharge and waste treatment. The food consumption of urban residents might significantly boost the food supply. Simultaneously, consumption behavior can dramatically affect food loss and waste [4]. Approximately one third of the food annually produced for human consumption is lost or wasted in China [53], while 40% of food-sourced nitrogen was thrown away as food waste in Xiamen [3].

In recent years, the Xiamen waste treatment system has been improving, gradually processing a mass of human excreta and food waste. Nevertheless, the common waste treatment system only alters the nitrogen released into different environmental media [4]. For example, landfill is the main treatment process for food waste in Xiamen, causing soil deposition. Nitrogen emission into the air caused by landfill is minimal, and so it can be ignored [54]. Incineration treatment is rare, and the discharge is generally into the air, with abundant water and organic matters contained in food waste. In terms of human excreta treatment, urine is gathered in the sewage treatment system through pipelines, and most sewage is discharged into the soil [52]. In addition, the amount of recycled food-sourced nitrogen, such as excreta, returned to fields was limited or even decreased because of reduced agricultural acreage and the mechanization of farming [4].

**Figure 2:**
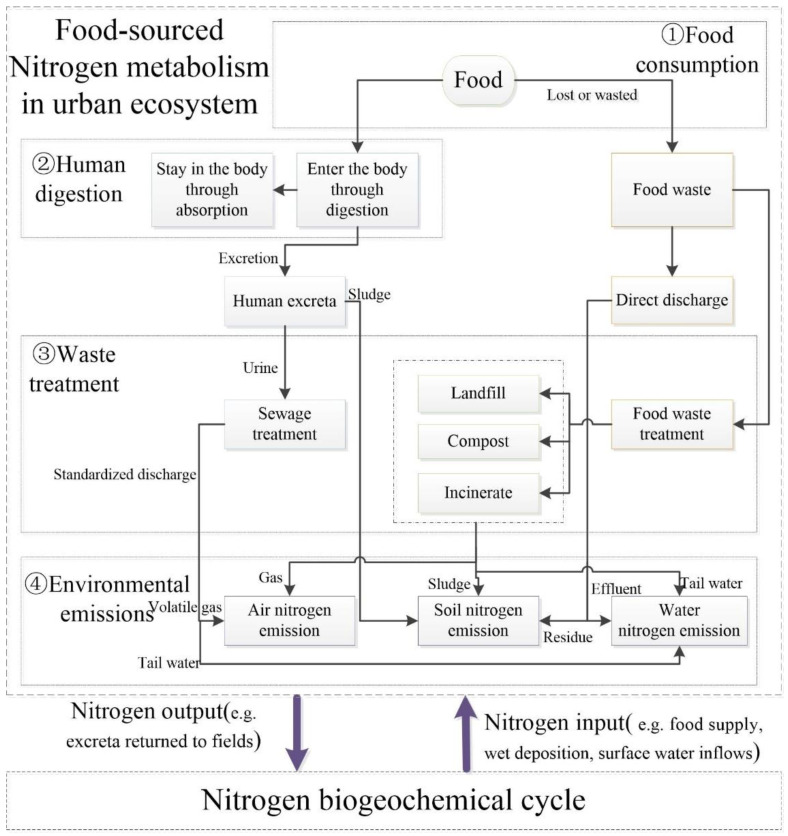
Flow path diagram of urban metabolism of food-sourced nitrogen.

In fact, we concentrated on artificial urban metabolism to explore the dynamic causal chains of food-sourced nitrogen in the process of urbanization, not including other inadvertent biogeochemical processes, such as food supply and wet deposition. Therefore, the urban metabolism of food-sourced nitrogen was shown in Figure 2. Among them, the calculation methods and parameters of each path are shown in Table A1 and Table A2.

#### 2.3.3. Impact Factor Analysis of Food-Sourced Environmental Nitrogen Emissions

A structural equations model (SEM) was applied for analyzing the factors influencing urban household environmental nitrogen emissions. This approach helps to understand the direct and indirect interactions between variables [55]. The discussions of fit in the SEM-related literature has led to various recommendations for the precise application of various fit indicators [50]. We used questionnaire reliability analysis, including Cronbach’s alpha and composite reliability (CR), to test the internal consistency of the overall questionnaire data, which is an important aspect of questionnaire data quality. We also used model fit indices like chi-square (χ^2^), goodness of fit index (GFI), root mean square residual (RMSR), root mean square error approximation (RMSEA), normed fit index (NFI), comparative fit index (CFI), incremental fit index (IFI), parsimony goodness of fit index (PGFI) and parsimony normative goodness index (PNFI), to verify whether the observed data support the validated theoretical model. Good model fits are also reflected by CR and AVE values above 0.5, RMR and RMSEA values below 0.08, GFI value above 0.9, NFI, CFI and IFI values above 0.95, and PGFI and PNFI values above 0.5.

## 3. Results

### 3.1. Household Food Consumption and Nitrogen Consumption

**Figure 3:**
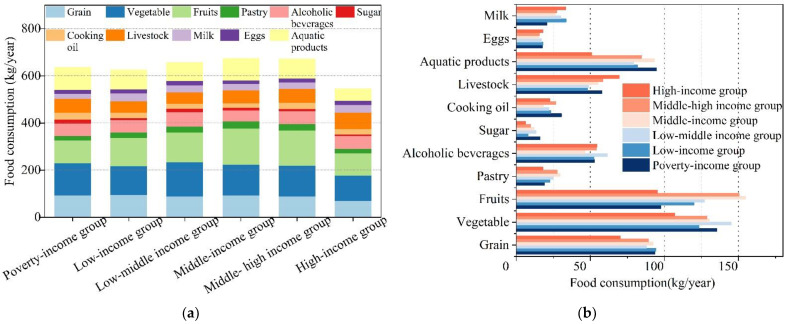
Per capita food consumption of six income groups households. (**a**) The total amount of per capita food consumption in six income groups households. (**b**) The types of per capita food consumption in six income groups households.

The average food consumption per capita from surveyed households was 624.28 kg/year. The six categories of income household consumption could be ranked in decreasing order as: middle-income group (674.3 kg/year) > middle–high-income group (672.9 kg/year) > low–middle-income group (656.9 kg/year) > poverty-income group (635.7 kg/year) > low-income group (625.6 kg/year) > high-income group (545.5 kg/year) (Figure 3a). Diets consisted mainly of vegetables, fruits, and grains. The order of food consumption per capita of the eleven types of food is as follows: vegetables (128.3 kg/year) > fruits (124.2 kg/year) > grains (87.9 kg/year) > aquatic products (80.7 kg/year) > livestock (56.4 kg/year) > alcoholic beverages (53.6 kg/year) > milk (28.6 kg/year) > cooking oil (23.9 kg/year) > pastry (23.7 kg/year) > eggs (16.9 kg/year) > sugar (10.9 kg/year) (Figure 3b).

In terms of food consumption quantity, the highest food consumption per capita household (middle-income group) was 17.2% higher than the lowest (high-income group). In terms of food consumption structure, surveyed households in Xiamen City consumed more plant-based foods (such as vegetables, fruits, grains) and less animal-based foods (aquatic products and livestock). The six income groups’ diets showed wide differences with respect to their respective proportions of the eleven types of food. For instance, fruit consumption was represented by an “inverted U” shape, that is, as household income increased, fruit consumption per capita first decreased and then increased; the peak value was 154.83 kg/year in the middle-income group. Egg consumption, by contrast, only varied slightly among households, at 17 kg/year (Figure 3b).

**Figure 4:**
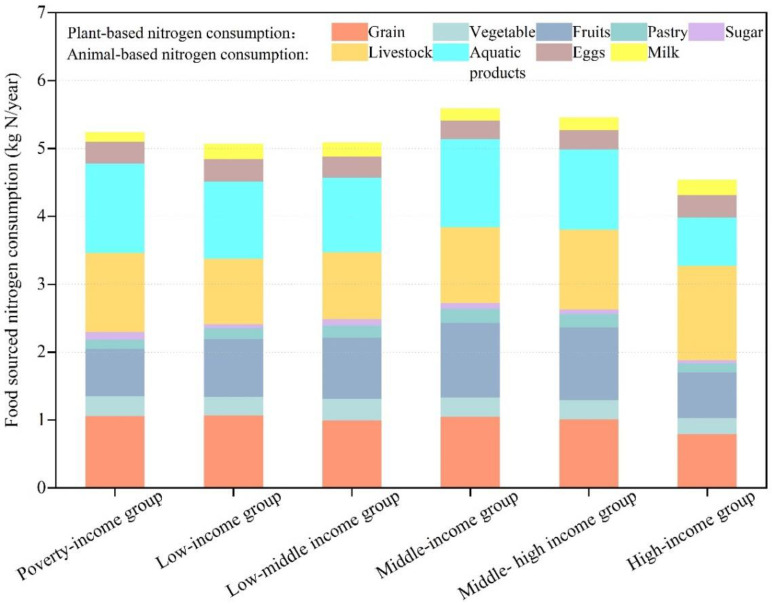
Per capita food-sourced nitrogen consumption for the six household groups.

The differences in nitrogen content meant that the order of food-sourced nitrogen consumption per capita for the six income categories of households did not exactly match food consumption (Figure 4): middle-income group (5.59 kg N/year) > middle–high-income group (5.46 kg N/year) > poverty-income group (5.24 kg N/year) > low–middle-income group (5.09 kg N/year) > low-income group (5.07 kg N/year) > high-income group (4.54 kg N/year). Given that livestock contain a higher proportion of nitrogen than other foods, the poverty-income group had the higher food-sourced nitrogen consumption per capita than the low-middle income group, in spite of its total food consumption per capita being lower than the latter’s.

Most of the nitrogen from household consumption was derived from aquatic products and livestock, which accounted for 43.7% of total food-sourced nitrogen. The order of the nine categories of food-sourced nitrogen consumption per capital are as follows: livestock (1.13 kg N/year) > aquatic products (1.12 kg N/year) > grains (0.99 kg N/year) > fruits (0.88 kg N/year) > eggs (0.31 kg N/year) > vegetables (0.28 kg N/year) > milk (0.19 kg N/year) > pastry (0.16 kg N/year) > sugar (0.08 kg/year). Here, nitrogen from cooking oils and alcoholic beverages were ignored, considering that the nitrogen content in such foods is extremely low.

### 3.2. Environmental Nitrogen Emissions Due to Household Food Consumption

**Figure 5:**
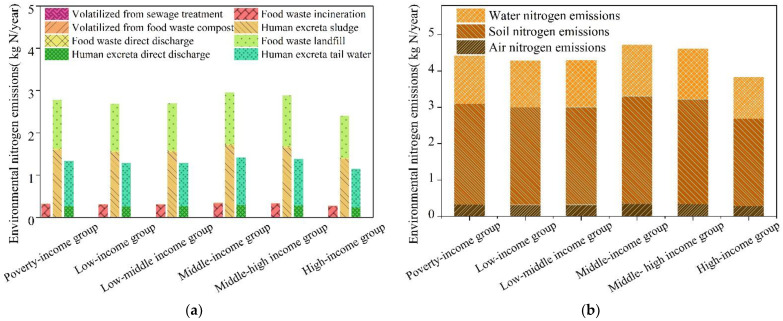
Environmental nitrogen emissions of the six income groups of households. (**a**) Environmental nitrogen emissions in the metabolism paths of the six income groups of households. (**b**) Environmental nitrogen emissions into air, soil, and water of the six income groups of households.

The results of environmental nitrogen emissions are shown in Figure 5. In the nitrogen flow paths, there were eight types of nitrogen metabolism and their emissions per capita were in the following order: human excreta sludge (1.607 kg N/year) > food waste landfill (1.160 kg N/year) > human excreta tail water (1.072 kg N/year) > food waste incineration (0.293 kg N/year) > human excreta direct discharge (0.271 kg N/year) > food waste direct discharge (0.032 kg N/year) > volatilized from sewage treatment (0.013 kg N/year) > volatilized from food waste compost (0.012 kg N/year) (Figure 5a).

In terms of environmental media classification, most nitrogen emissions due to household food consumption enter into the soil, including human excreta sludge, food waste landfill, and food waste direct discharge, which accounted for 62.8% of total nitrogen emissions. Water emissions, including human excreta direct discharge, and human excreta tail water, accounted for 30.1% of total nitrogen emissions. The air emissions, including food waste incineration, volatilized from sewage treatment and food waste compost, respectively, only accounted for 7.1% of total nitrogen emissions (Figure 5a).

In terms of the six income groups for households, food-sourced nitrogen emissions per capita were: middle-income group (4.72 kg N/year) > middle–high-income group (4.61 kg N/year) > poverty-income group (4.43 kg N/year) > low–middle-income group (4.30 kg N/year) > low-income group (4.28 kg N/year) > high-income group (3.83 kg N/year) (Figure 5b). The order of the emission intensities per capita for the six income groups’ food consumption activities was the same as for food-sourced nitrogen consumption. In household food consumption, 84.5% of the food-sourced nitrogen was discharged into the air, soil, and water through direct discharge and waste treatment.

### 3.3. Key Factors Influencing Food-Sourced Environmental Nitrogen Emissions

We used SPSS22.0 to analyze the reliability of questionnaire data. The internal consistency for our scale, as assessed by Cronbach’s alpha, was 0.847 (>0.8). In addition, the CR value of all variables also remained within a reasonable range (>0.5). They both showed the questionnaire data to have good reliability. Then, we used AMOS22.0 for confirmatory factor analysis and path analysis. The hypothesized path model yielded a good fit to data, as shown by a χ^2^ value (χ^2^/df = 3.174 < 5) and a GFI = 0.954 > 0.9, and other results fitting the optimization index and acceptance criteria, as shown in Table 4.

**Figure 6:**
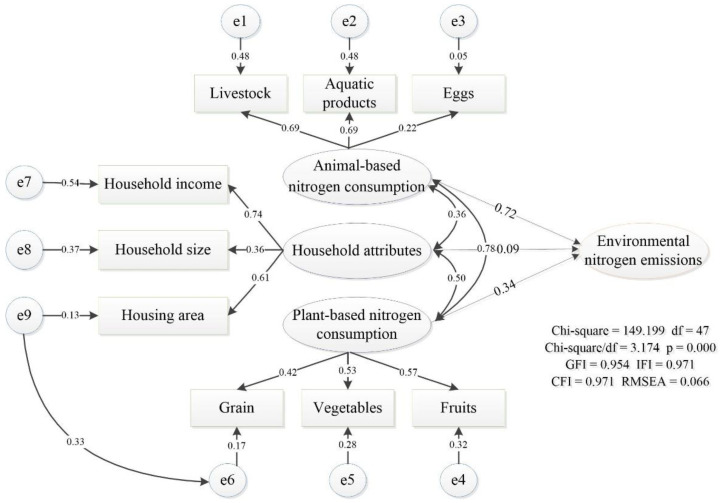
Influencing mechanism of key factors on the food-sourced environmental nitrogen emissions of the six income groups of households.

The final model and standardized results were listed in Figure 6. Squares represented observed variables and ellipses represented unobserved variables evaluated by observed variables. Standardized path coefficients were shown in the middle and represent partial regression coefficients. Whole following factors had positive effects on environmental nitrogen emissions. The path analysis illustrated that animal-based food consumption had the strongest positive effect on environmental nitrogen emissions, followed by plant-based food consumption and household attributes. In terms of the absolute values of standard path coefficients, the sequence of effects was livestock, aquatic products, fruit, vegetables, grain, eggs, household income, housing area, and household size. Moreover, household attributes, plant-based food consumption, and animal-based food consumption were mutually connected and should be considered as highly interactive. There was a significant correlation between household attributes and plant-based food consumption (path coefficient = 0.50).

## 4. Discussion

### 4.1. Characterizing Environmental Nitrogen Emissions from Urban Household Food Consumption

The dietary habits of Xiamen residents are built by material, economic, and cultural factors, which may affect environmental nitrogen emissions in various ways [56]. The questionnaire survey can directly link residents’ dietary habits to nitrogen emissions from food consumption and help to propose a breakthrough for nitrogen-reduction policymaking. Currently, household greenhouse gas emissions were mostly dependent on housing areas; it is believed that less densely populated housing areas have lower emissions [57,58]. However, larger housing areas would result in smaller nitrogen emissions on the condition that other factors were held constant in our study, so policies to ensure spacious housing per capita might reasonably be an effective action to reduce environmental nitrogen emissions for Xiamen City.

Household consumption will play a more and more important role in shaping China’s food demand and nitrogen emissions in future. It might be necessary to know that the tendencies of Chinese urban lifestyles are in order to realize the development of a “clear your plate” campaign. We did find household size to be a major factor influencing environmental nitrogen emissions, and larger households tend to be more efficient in terms of food consumption per capita. The Chinese government implementation of a “three-child policy” provides a possible way for reducing environmental nitrogen emissions.

In our study, the nitrogen emissions gradient existing among the six income groups of households can provide worthwhile information for Chinese urban households in the near future. In 2011, most urban households in Xiamen City had low or medium incomes below 20,000 yuan/month and higher nitrogen emissions than the high-income group (over 20,000 yuan/month). Future urbanization and socioeconomic development will bring about the improvement of household income levels [59], an increase in housing area, and the expansion of household size. Therefore, the proportion of high nitrogen emission households will gradually decrease, while low nitrogen emission households might increase slowly. In addition, environmental nitrogen emissions did not show a strictly inverted ‘U’-shape as household income increased, that is, the Environmental Kuznets Curve (EKC) [60], and they merit further study.

### 4.2. Policy Making toward Urban Food Security Consumption Patterns

Policymaking for a city’s food security must take comprehensive measures in terms of policy scope, priority, and implementation time. In the case of Xiamen, the policy scope should encompass the entire path of urban metabolism of food-sourced nitrogen, including food consumption, human digestion, waste treatment, and environmental emissions. Specific policies should include reducing pre-meal food waste and “cleaning your plate” at the food consumption path, eating the right amount, and exercising after meals to maintain digestion and absorption, raising waste treatment rates at the waste treatment path, and improving nitrogen recovery rates along the environmental emissions path.

Policy priority should be given to the consumption of livestock and aquatic products, as this causes the maximum environmental nitrogen emissions. For example, halving the consumption of meat, dairy products, and eggs would achieve a 40% reduction in nitrogen emissions [61]. Due to the large disparity in nitrogen emissions profiles among different income households, high-nitrogen households (such as the middle-income group and the middle–high-income group) should be targeted for policy-driven lifestyle adjustments. In addition, technological breakthrough in high-nitrogen path, such as human excreta sludge, food waste landfill, and human excreta tail water, could advance nitrogen reduction. It is well known that urban human excreta are today usually considered as waste to be disposed [62,63].

Implementation times should be based on predictable changes in urban development and pay attention to household consumption, which is predicted to increase remarkably in the coming years. Control of food waste to reduce nitrogen emissions due to household food consumption is most urgent, followed by updating technology in urban waste treatment plants, developing good lifestyle habits, such as avoiding impulse-buying and bulk purchases, and encouraging the use of leftovers within safety dates.

### 4.3. Technologies Integration and Innovation toward Sustainable Nitrogen Cycling

Technologies for sustainable nitrogen cycling in the urban ecosystem can be categorized as loss reduction technology and recycled use technology [4]. Loss reduction technology aims to minimize nitrogen loss, including reducing food waste and wastewater direct emissions. For example, Zhu et al. introduced Feammox, a new microbial process that linked the nitrogen cycles, to effectively transform ammonium to dinitrogen gas, and remove nitrogen from various polluted water sources in municipal wastewater treatment [64]. Ma et al. proposed that a third-generation anaerobic reactor could be a revolutionary breakthrough for food waste treatment, with high bioenergy conversion efficiency. [65].

Recycling technologies seek to form recovery and reuse loops, including efficiently collecting food waste and wastewater to transfer into fertilizer. Wan et al. found that co-digestion of food waste, wastepaper, and plastic in a new single stage rotary anaerobic reactor can enhance the recovery of ammonium-N and simultaneously produce energy (biogas) [66]. Huang et al. used air stripping in struvite decomposition and the simultaneous removal and recovery of nitrogen from different types of waste water to minimize environmental impacts of excess nitrogen [67,68]. In addition, nitrogen contained in biosolids (known as sewage sludge) can be recycled to the land, given that it has been treated or ‘stabilized’ at dilute initial ammonia concentrations [69].

The biodegradable fraction of food waste and wastewater is generally recognized as a sustainable source for nitrogen cycling in urban ecosystems. Anaerobic digestion is the most used process in many countries [70,71], especially in China, accounting for 76.1% of all food waste pilot projects [72]. The majority of anaerobic digestion facilities for bio-waste currently operating in the EU28 can play an important role for the implementation of EU policy regarding renewable energy and waste management [73]. At the same time, China has the greatest interest in this technology compared to other countries, which carries the potential to achieve economic and environmental benefits [74,75]. However, urban anaerobic digestion pilot facilities are facing problems of under-capacity, inefficient biogas utilization, and other issues, in China and in other developing countries [76,77,78]. The promotion of synergies between mitigation and recycling technologies to provide urban systemic benefits and ensure high metabolic efficiency is to be desired.

## 5. Conclusions

As cities have become the primary habitat for humans, nitrogen emissions due to household food consumption have become increasingly significant. In this study, we presented a quantitative model for simulating the urban metabolism of food-sourced nitrogen and applied it in the case of Xiamen City, China based on a large sample onsite survey. Surveyed household food consumption per capita was 624.28 kg/year, significantly higher than the 386.9 kg/year of average Chinese urban residents. With regard to dietary structure, the diets of the surveyed households were more plant-based (vegetables, fruits, grains) and less animal-based (aquatic products, livestock and eggs), which follows a similar track as developed countries. Most of the food-sourced nitrogen derived from aquatic products and livestock, which accounted for about half of total food consumption. Further studies will be needed to quantify the nitrogen reduction potentials in each consumption category (e.g., fish, pork, and beef) based on existing technology and evaluate practical feasibility.

Most of the food-sourced nitrogen is discharged into the air, soil, and water through direct discharge and waste treatment. Loss reduction and recycling technology should be the focus of sustainable nitrogen cycling in China. Considering that household income was the main factor of environmental nitrogen emissions, there was a large disparity in nitrogen emissions among the six household groups. High-nitrogen households, such as the middle-income group and middle–high-income group, should be the target of policies to promote lifestyle adjustments. Moreover, the policy scope for cities’ food security should cover the entire path of urban metabolism and aim to reduce pre-meal food waste and promote “cleaning your plate”, eating the right amount and exercising after meals.

Our evaluation is limited to nitrogen emission related to household food consumption and does not consider emissions from household energy use, etc. The urban metabolism of food-sourced nitrogen emission flow paths and accounting methods developed in this study can be readily applied to other cities. They provide helpful tools to comprehend and analyze different income groups of urban households, and can be used to develop targeted policies for food-sourced nitrogen emissions reduction. We suggest that different food categories and production sources, household willingness and activity in waste disposal, and waste treatment technology innovation and integration, should be comprehensively considered in future urban nitrogen cycling studies.

## Figures and Tables

**Table 1:** Components and survey variables in questionnaire.

Latent Variables	Observed Variables
Household attributes	Household size, household income, household structure, housing area, building age
Household food consumption	Plant-based food: grain (including rice, miscellaneous grains, and flour), vegetables, fruits, edible oils, alcoholic beverages, pastry, sugar, tea; Animal-based food: livestock (pork, beef, sheep, poultry), eggs, aquatic products (fish and seafood), milk

**Table 2:** The transformation standards and occurrence frequencies of respondents’ profiles.

Observed Variables	Categories	Transform Standards	Occurrence Frequency (%)
Household size	One person	1	3.59
	Two people	2	19.96
	Three people	3	51.10
	Four people	4	15.97
	Five people	5	9.18
	Six people	6	0.20
Household income (RMB yuan/month)	<2500 (poverty-income)	1	19.16
	2500–5000 (low-income)	2	26.55
	5000–7500 (low–middle-income)	3	26.95
	7500–10,000 (middle-income)	4	12.97
	10,000–20,000 (middle–high-income)	5	12.57
	>20,000 (high-income)	6	1.80
Household structure	Only the elderly (over 65 years old)	1	4.19
	Containing adults and minors (0–17 years old)	2	37.33
	Containing adults and the elderly	3	9.38
	Containing adults, the elderly and minors	4	6.79
	Three adults (18–64 years old)	5	36.13
	Three or more adults	6	6.19
Housing area	<39 m^2^	1	5.19
	40~69 m^2^	2	20.56
	70~89 m^2^	3	27.54
	90~119 m^2^	4	29.94
	120~149 m^2^	5	11.58
	>149 m^2^	6	5.19
Building age	Before 1985 (old-style community)	1	23.75
	1985–1989 (old-style community)	2	35.33
	1990–1994 (transitional community)	3	24.35
	1995–1999 (transitional community)	4	4.99
	2000–2005 (commercial community)	5	6.59
	After 2005 (commercial community)	6	4.99

**Table 3:** The nitrogen conversion coefficients of household food consumption ^1^.

Items	Nitrogen Content/%	Items	Nitrogen Content/%
Grain	1.13	Sheep	2.19
Vegetable	0.22	Poultry	1.89
Fruits	0.71	Aquatic products	1.39
Pastry	0.71	Milk	0.68
Pork	1.77	Eggs	1.83
Beef	2.20		

^1^ Note: The data were referred from FAO: http://www.fao.org/faostat/en/#data/FBS.

**Table 4:** Fitting optimization index of SEM.

Item	Indices	Acceptance Criteria	Fitting Result
Absolute fitting indicators	χ^2^/df	<5	3.174
	GFI	>0.9	0.954
	RMR	<0.08	0.060
	RMSEA	<0.08	0.066
Value-added fitting index	NFI	>0.95	0.958
	CFI	>0.95	0.971
	IFI	>0.95	0.971
Simplified fitting index	PGFI	>0.5	0.575
	PNFI	>0.5	0.682

## Data Availability

The data presented in this study are available on request from the corresponding author.

## Appendix A

**Table A1:** Calculation formulas of food-sourced nitrogen metabolism and environmental nitrogen emissions.

Items	Variables	Formulas
Food consumption	N in food waste	Per capita food-sourced nitrogen consumption × food waste ratio
Human digestion	N in human absorption	Per capita food-sourced nitrogen consumption × human food absorption ratio
	N of human excreta	Per capita food-sourced nitrogen consumption × human excreta emission ratio
Waste treatment	N in food waste treatment	N in food waste × percentage of garbage treatment with standard
	N in human excreta treatment	N of human excreta × percentage of sewage treatment of city
	N volatilized from sewage treatment	N in human excreta treatment × N2O emission factor
	N in food waste compost	N in food waste treatment × composting ratio of food waste treatment
	N in food waste landfill	N in food waste treatment × landfill ratio of food waste treatment
	N in food waste incineration	N in food waste treatment × incineration ratio of food waste treatment
	N volatilized from food waste compost	N in food waste compost × fertilizer volatile ratio
Environmental emissions	N in food waste direct discharge	N in food waste—N in food waste treatment
	N in human excreta direct discharge	N of human excreta—N of human excreta sludge—N of human excreta tail water—N emissions from sewage treatment
	N of human excreta sludge	N in human excreta treatment × N removal ratio of human excreta
	N of human excreta tail water	N in human excreta treatment × (1-N removal ratio of human excreta)
	N emissions in soil	N in food waste direct discharge + N of human excreta sludge + N in food waste landfill
	N emissions in water	N in human excreta direct discharge + N of human excreta tail water
	N emissions in air	N emissions from sewage treatment + N volatilized from food waste compost + N in food waste incineration

**Table A2:** Required parameters for calculating environmental nitrogen emissions.

Items	Parameters	Unit	Values	Source
Food consumption	N content in food waste	%	40	[3]
Human digestion	Human food absorption ratio	%	2	[79]
	Human excreta emission ratio	%	88	[80]
Waste treatment	Percentage of garbage treatment with standard	%	98.32	[81]
	Percentage of sewage treatment of city	%	90.40	[81]
	N removal ratio of human excreta	%	60	[82]
	The composting ratio of food waste treatment	%	2.6	[83]
	The landfill ratio of food waste treatment	%	61.4	[83]
	The incineration ratio of food waste treatment	%	15.9	[83]
Environmental emissions	N volatilized ratio from human excreta	%	24.5	[84]
	N2O emission factor	kg N2O/kg N	0.005	[85]
